# Developing health research capacity and capability in underserved geographies: a case study from a new medical school

**DOI:** 10.1186/s12961-026-01452-x

**Published:** 2026-03-09

**Authors:** Jo-Anne Johnson, Georgia Winnett, Sanjiv Ahluwalia

**Affiliations:** School of Medicine, Anglia Ruskin University, Bishop Hall Lane, Chelmsford, Essex CM1 1SQ United Kingdom

**Keywords:** Research equity, Health inequalities, Clinical academic training, Research inclusion, Capacity building, Sustainable research infrastructure, Medical education, Undergraduate and postgraduate research opportunities

## Abstract

**Background:**

Research-active healthcare institutions are associated with improved patient outcomes and staff satisfaction. However, research funding in the United Kingdom remains disproportionately concentrated in established academic centres, limiting opportunities for newer institutions – often located in regions with greater health need – to develop research capacity. This entrenches health inequalities and restricts the pipeline of clinical researchers in underserved areas.

**Methods:**

We used a case study methodology to explore how one new United Kingdom medical school, situated within a teaching-focused university and region of relative socioeconomic disadvantage, built research capacity and supervisory infrastructure from the ground up. Drawing on internal expertise and infrastructure, strategic partnerships and national funding schemes, we examined the structural enablers and barriers encountered in establishing a locally relevant research ecosystem.

**Results:**

A phased approach to capacity building was employed, starting with internal resources and strategic collaborations. Supervisory infrastructure was developed through networked partnerships, enabling undergraduate and postgraduate research opportunities. The creation of thematic research groups evolved into recognized research centres. This foundation enabled successful bids for competitive external funding, including undergraduate and postgraduate research schemes, which in turn developed research capacity. We highlight how equitable access to research opportunities – particularly for students from widening participation backgrounds – was embedded within the curriculum and supported by funded placements. Our experience demonstrates that early, targeted investment in research infrastructure, even in settings with low baseline research activity, can generate sustainable capacity, increase participation and reduce regional disparities in research engagement.

**Conclusions:**

To promote equity in research funding and reduce health inequalities, national funding bodies should adopt more inclusive investment strategies that actively support emerging centres. Structural reform is needed to ensure that funding mechanisms do not solely reward existing capacity but also foster its development in underserved regions. Our findings offer a scalable model for building sustainable research ecosystems in new or underfunded centres, aligned with local health needs and population outcomes.

**Supplementary Information:**

The online version contains supplementary material available at 10.1186/s12961-026-01452-x.

## Background

It is well established that research-active National Health Service (NHS) trusts achieve better patient outcomes [1–3] and lower mortality rates [2, 4, 5]. These improvements may stem directly from research findings, for example, participation in clinical trials has been associated with 20–25% improved clinical outcomes in women’s health [1], or indirectly through improved adherence to evidence-based practice, and improved staff recruitment and retention [3, 5]. Patients involved in research also report higher satisfaction levels, including increased confidence in healthcare professionals and a stronger sense of involvement in their care [6].

Many healthcare professionals recognize the value of research and are keen to contribute [7, 8]. However, barriers such as a lack of protected time often prevent participation. Findings from the 2023 Royal College of Physicians (RCP) census show that over a third of doctors (36%) want to be involved in research, but the main barrier is the absence of ringfenced time in job plans [9]. Consequently, roles that incorporate research activity are especially attractive. In addition, staff who are actively engaged in research report greater job satisfaction [10].

We propose a definition of research equity as being the fair and inclusive distribution of research opportunities, funding, infrastructure and outcomes across geographic, institutional and demographic boundaries. It ensures that all populations, regardless of location, socioeconomic status or organizational affiliation, can benefit from and contribute to research. Achieving research equity is essential not only for reducing health inequalities but also for building a sustainable and representative research workforce across the NHS. This definition is informed by the equity-oriented research production paradigm [11] and equity science frameworks [12].

A major challenge in United Kingdom medicine is the persistent inequity in research funding and infrastructure. Traditional funding models disproportionately favour well-established academic centres, typically large research-intensive universities, while smaller or less research-active institutions remain underfunded and under-resourced. For example, in 2021–22, just five higher education institutions received over 40% of total UK Research and Innovation (UKRI) funding [13], the United Kingdom’s main national research funder for academic research across all disciplines (including medicine, science, engineering, arts and humanities). This concentration of investment leaves many NHS trusts, particularly those serving more deprived or rural populations, without the support needed to build sustainable research capacity [14].

This research inequity contributes to broader health inequalities. Regions with low research activity are often the same areas facing poorer health outcomes and higher levels of deprivation [15, 16]. When research is concentrated in specific geographic or institutional pockets, the needs of underrepresented populations are less likely to be studied or addressed. As noted by the National Institute for Health Research (NIHR), the United Kingdom government’s national funder of health and social care research, improving local research delivery is essential for tackling health disparities and ensuring evidence-based interventions are relevant to diverse populations [17].

Furthermore, this imbalance is counterintuitive at a time when the United Kingdom is facing a critical shortage of clinicians entering research careers [17]. Without a more equitable distribution of research funding and support, the pipeline of clinician–researchers will continue to falter, particularly in regions and trusts most in need of development.

Addressing research inequity requires a deliberate shift in how we fund, support and conceptualize research across the NHS. Rather than concentrating investment in already research-active centres, we must build capacity in underrepresented regions and institutions.

However, funding still largely follows existing research activity, making it difficult for new centres to gain a foothold. There are more accessible schemes emerging such as The NIHR’s Research for Patient Benefit programme [18] which supports locally driven, applied research. But even with these more accessible schemes, uptake remains skewed towards established academic sites with strong research track records [13].

To break this cycle, proactive, place-based strategies are needed to nurture research culture and capability where it does not yet exist. Evidence shows that with the right support, research activity can flourish in new environments, improving local care and clinician engagement [19].

Many new medical schools are established in regions marked by socioeconomic disadvantage and health inequalities – areas that present compelling opportunities for research that directly addresses local patient needs [20]. For example, areas facing social and economic deprivation consistently face challenges with healthcare accessibility, lower health literacy and health-related knowledge, adverse social determinants of health and healthcare workforce shortages [16, 21, 22]. However, newer medical schools are typically affiliated with universities and healthcare providers that have limited research infrastructure or a history of low research activity [20]. This creates a paradox: while the need for context-specific research is acute, the capacity to deliver it is often lacking.

This case study examines how Anglia Ruskin University, a new medical school in the United Kingdom, has developed research, with the aim of addressing the research question: How can health research capacity and capability be developed in geographies historically underserved by research, in pursuit of a more equitable research ecosystem?

## Methods

### Methodology

In this case study, we explore how research capacity can be developed in a research-underserved setting, at a new United Kingdom medical school. We adopted a single-case, exploratory, and instrumental case study design [23]. The exploratory approach was appropriate given the absence of established models for developing research capacity in new medical schools and underserved regions. An instrumental design was chosen as the case is not examined solely for its intrinsic value, but to illuminate the broader issue of research inequity in the United Kingdom. This methodology enabled us to analyse the processes, enablers and barriers encountered, and to draw transferable lessons for other contexts facing similar challenges.

### Data collection

Institutional records were the primary data source, including strategic plans, administrative records of research infrastructure development, programme documentation for undergraduate and postgraduate research opportunities and outputs such as conference presentations and publications. We also reviewed records from internal governance processes (e.g. research steering group reports) and relevant external policy guides and funding applications (e.g. National Institute for Health and Care Research/NIHR Integrated Academic Training/IAT scheme). Together, these sources enabled us to capture both the internal development of research capacity and the external enablers that shaped this trajectory.

### Data analysis

**Fig. 1:**
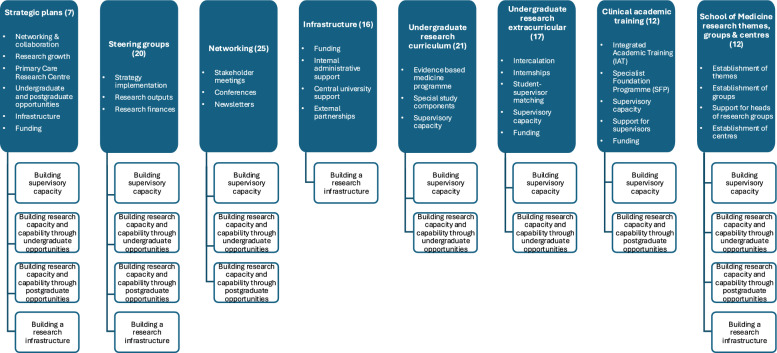
Development of Themes from Documentary Data**.** Categories of documentary data (blue boxes – headings, with number of documents reviewed in brackets) were grouped into provisional themes (blue boxes – bullet points) using inductive coding. These were then synthesized into the final overarching themes (white boxes)

We employed an explanation-building approach [24] to iteratively develop a narrative linking institutional activities with outcomes in research capacity-building. A total of 130 institutional records and documentary sources were systematically reviewed, and categories of data were coded into emerging provisional themes. These provisional themes reflected both barriers and enablers of research development and were refined through collaborative discussion between the authors to produce the final themes (Fig. 1).

The analysis was primarily inductive, allowing themes to emerge from the data itself rather than fit the data into pre-existing coding frames [25], though informed deductively by the research question and policy concerns on research capacity-building and equity. Whereas inductive analysis prioritizes discovery, deductive analysis ensures that the inquiry remains connected to existing debates and policy priorities**.** Iterative refinement ensured that themes were both grounded in the local case and transferable to broader contexts of research development in underserved geographies. Case vignettes are used to illustrate each theme.

**Fig. 2:**
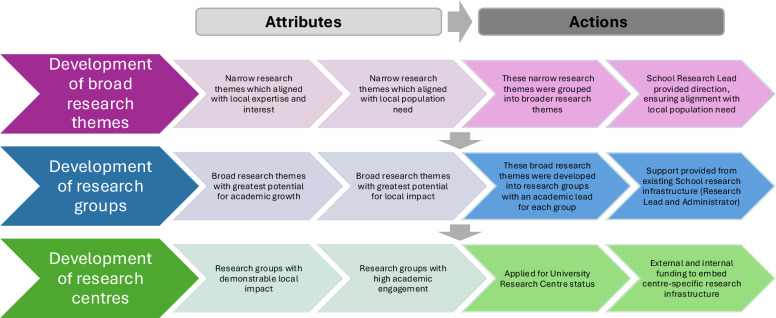
Developing research themes and centres at a new United Kingdom medical school**.** Phased development of research themes, groups, and centres at a new United Kingdom medical school situated in a poorly research-resourced setting

**Fig. 3:**
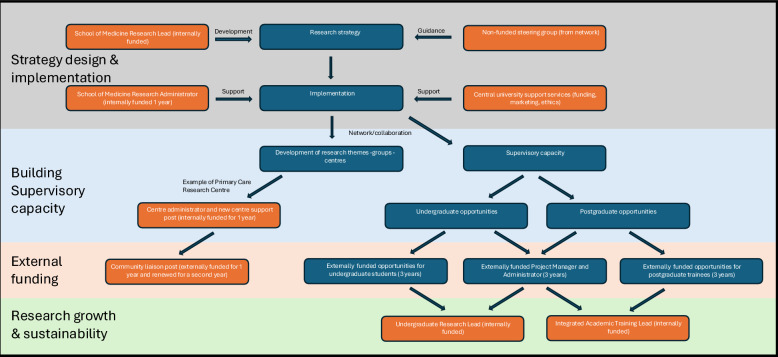
A phased approach to developing research capacity, capability and infrastructure**.** A phased approach to building sustainable research within a new medical school located in a region historically underserved by research. The four labelled phases are represented by the four background colours. The blue boxes represent activities undertaken within each of the phases. The orange boxes represent infrastructure development

**Fig. 4:**
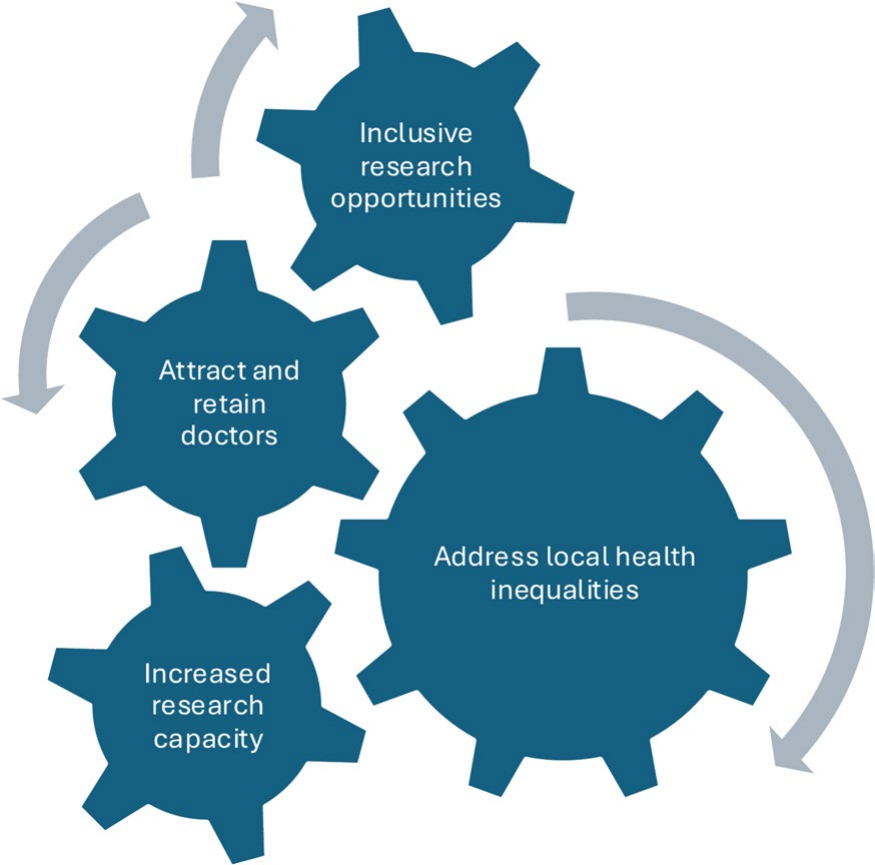
Positive feedback loop linking inclusive research opportunities, workforce retention and increased research capacity to address local health inequalities. A conceptual model of the positive feedback loop that underpins sustainable research capacity building in new and underserved clinical research settings, with direct implications for reducing health inequalities

Figure 2 represents a data-driven model that emerged from our inductive analysis of institutional development phases. These phases in turn are illustrated in Fig. 3. In contrast, Fig. 4 is a conceptual model developed during the discussion phase to illustrate broader implications and transferable learning.

## Results

The results of the thematic analysis identified emerging provisional themes from the categories of documentary data reviewed (Fig. 1). These were then refined to the final four overarching themes – building supervisory capacity, building research capacity and capability through undergraduate opportunities, building research capacity and capability through postgraduate opportunities and building a research infrastructure. 

### Theme 1: building supervisory capacity

Developing robust research supervisory capacity was central to the sustainable growth of research at Anglia Ruskin University (ARU) School of Medicine, a new medical school in Essex, United Kingdom.

However, as is the case with many newer medical schools embedded in teaching-focused universities or non-research-intensive healthcare trusts, expertise is dispersed and often limited in any one location. High-quality, directive supervision is essential yet cannot be delivered through traditional models that rely on a critical mass of local senior clinical researchers. The response to this challenge at ARU has been to adopt a collaborative and networked approach to supervisory development.

Case study vignette – a collaborative approach to building supervisory capacity Essex is a region in the United Kingdom with areas of high socioeconomic deprivation [26], particularly within its coastal regions, and health inequalities. ARU School of Medicine was set up with the aim of addressing clinician shortages within Essex [27] and indirectly improving the healthcare outcomes/reducing health inequalities of the local population.

ARU welcomed its first cohort of 100 medical students in 2018 and has graduated three cohorts of doctors to date. Anglia Ruskin University itself is recognized for its commitment to high-quality teaching and widening participation. While this educational focus has historically meant a more limited research infrastructure compared with research-intensive institutions, it has also created space for innovation and purposeful research development.

In 2022, the School of Medicine developed a 3-year research strategy (Supplementary files 2 and 3) focused on building supervisory expertise and clinical academic training, with the overarching goal of enhancing healthcare research capacity and capability in Essex.

Networking initiatives were undertaken to connect the School with the wider university and clinical community to find local expertise and interest. From these, narrow research themes were derived which were grouped into several broader research themes aligning with the School’s ambition to improve health outcomes for the local population (Supplementary file 1). These themes provided a flexible framework to pool expertise and resources while allowing direction and focus to evolve over time.

These broad themes were subsequently reviewed by the School of Medicine Research Steering Group. Those themes deemed as having the greatest potential for impact on the health needs of the local population, as well as academic growth (as determined by available academic expertise within the School and wider university), were developed into structured research groups. These included Medical Education, Anatomy and Primary Care, with an academic lead allocated for each group, as well as mentorship from established academic leads from the wider university. These groups created supervisory capacity at the School for undergraduate medical student research.

Groups that demonstrated significant impact and academic engagement have since evolved into recognized research centres. Through internal/external funding, these centres have their own research infrastructure and postgraduate as well as undergraduate supervisory capacity. A key example is the Primary Care Research Centre, which has developed in response to both high unmet healthcare needs and strong internal and external support.

The analysis identified that this collaborative approach not only expanded supervisory capacity within neighbouring established research centres and institutes within the wider University and healthcare trusts but also enabled the pooling of expertise to lay the groundwork for emerging research themes within the School, strategically aligned with the school’s objectives. By embedding leadership and building infrastructure, the most impactful of these themes have evolved into formal research groups and centres each contributing to the growth of supervisory capacity (Fig. 2).

As the School’s research network continues to grow beyond Essex, there remains a commitment to forging new collaborative partnerships, broadening and deepening supervisory capacity and strengthened reputation in the research landscape.

### Theme 2: building research capacity and capability through undergraduate opportunities

At ARU School of Medicine there has been a strong focus on developing research training and opportunities for undergraduate medical students, recognizing that they represent the future clinical research workforce and that many are likely to remain in the local area after graduation. At ARU, approximately 60% of the medical student intake is drawn from the local area, and 51% of the most recent graduating cohort took up foundation posts within Essex.

Case study vignette – undergraduate research opportunities ARU School of Medicine integrated an “Evidence-Based Medicine” (EBM) theme across all 5 years of the undergraduate MBChB programme. This was in response to the General Medical Council (GMC), the regulatory body of doctors in the United Kingdom, stipulating that newly-qualified doctors must be able to apply scientific method and approaches to medical research, and integrate these with a range of sources of information used to make decisions for care [28].

The EBM theme provides the theoretical framework for the Special Study Components (SSCs). These SSCs incorporate an element of student choice, guiding learners through a research journey: from conducting a basic literature review in year 1, to formulating a research hypothesis in year 3 and culminating in a 9-month longitudinal research project in year 4. The research areas/supervisory capacity for the SSCs are drawn from the expanding research network, as well as growing research within the school. Over the 2-year period between March 2023 and March 2025, 17% of ARU medical students published in peer-reviewed journals, comparing favourably with the national average of 10–14% of medical students publishing at least one article throughout their medical degree [29].

A student-supervisor matching scheme was introduced outside the formal SSC structure. This flipped model allows students to propose their own research interests, with students then matched with appropriate supervisors from across the university and local NHS trusts. In the first year of implementation, 40 students were successfully matched, significantly widening access to research engagement beyond the standard SSC offerings.

Utilizing the developing supervisory capacity, the School has created student research opportunities across a diverse range of settings. These include both university- and trust-based environments, all underpinned by collaboration with the School.

Case study vignette – externally funded undergraduate research opportunities In 2023, the NIHR launched a closed funding call for new medical schools (Medical Schools Intercalated Degree and Internship/NMSIDI). This programme aimed to provide funded research opportunities for medical students, with a particular emphasis on supporting students from socioeconomically disadvantaged backgrounds.

Following the success of the bid, ARU School of Medicine drew on its existing networks and supervisory capacity to develop a range of extracurricular undergraduate research internships across the university and with local healthcare providers. They also secured university approval for 10 local, pre-existing master’s-level programmes for intercalation. Intercalation involves students taking an additional year out of their medical degree to study a separate degree qualification, and previously ARU medical students could only intercalate at external universities.

This NIHR funding provided full tuition fee coverage and stipends for 30 intercalated degrees, alongside stipends for 45 research internships over a 3-year period. Priority was given to students from socioeconomically disadvantaged (widening participation) backgrounds.

The NMSIDI funding bid recognized that many medical students undertake paid work to fund their studies [30], particularly students from socio-economically disadvantaged backgrounds, making extracurricular research and intercalation less accessible. It also recognizes that newer medical schools typically have a higher proportion of widening participation students, as is the case with ARU where 20–30% of their student intake meets widening participation criteria upon entry.

By first developing a foundation of undergraduate research activity and supervisory provision using existing networks and institutional resources, ARU positioned themselves in a strong position to compete for this external funding bid which provided financial support for medical students undertaking local extracurricular research and intercalated degrees.

### Theme 3: building research capacity and capability through postgraduate opportunities

ARU recognized that as well as undergraduate research opportunities, creating postgraduate research posts are vital in the development of local sustainable clinical research. In 2023, for the first time, the cohort of new medical schools established post-2018 were eligible to apply to the national NIHR IAT scheme.

Case study vignette – externally funded postgraduate opportunities The NIHR Integrated Academic Training (IAT) scheme provides a structured pathway for clinical trainees to engage in research alongside their clinical training, with protected and funded research time [31]. NIHR Academic Clinical Fellowships (ACFs), a core component of IAT, are targeted at pre-doctoral clinical trainees and offer 25% protected time for research over a 3-year appointment.

In 2023 ARU successfully bid for and recruited to four ACF posts. Success in the subsequent 2025 bid further reflects the School’s growing research activity, expanding networks and strengthened supervisory capacity.

In parallel, ARU has secured ongoing funding from NHS England for 12 Specialist Foundation Programme (SFP) posts in research each year [32]. These posts provide newly qualified foundation doctors with structured early exposure to research alongside clinical training.

ARU has drawn on expertise within its research network, including neighbouring medical schools to provide research supervision for these trainees as well as a coordinated, regional approach to postgraduate clinical research training.

A critical enabler of the success of ARU in the IAT bids has been the research supervisory infrastructure developed through their extended academic network. IAT bids are evaluated primarily on the strength and track record of the research groups in which trainees are embedded [31]. ARU therefore strategically centred their applications on the most established research centres and institutes within the University, particularly those aligned with NIHR priority areas.

At ARU a broader approach has been taken to supervision of SFPs, to reflect the earlier stage of their training, embedding these trainees in growing research centres within NHS trusts as well as more established centres within the University.

As well as their potential as future leaders in clinical research, there is a recognition that these trainees provide vital research capacity as they progress along their journey. As such, ARU aims to place more trainees in research centres within the School of Medicine itself, helping to develop research which aligns with local healthcare priorities.

### Theme 4: building a research infrastructure

Before late 2022, the School had no formal research infrastructure. The guiding principle was to start with existing internal resources and then strategically use external funding to expand capacity as research activity increased.

Case study vignette – building a research infrastructure The School utilized internal funding to appoint a School of Medicine Research Lead to design and implement a research strategy, and this was supported by an internally-funded research administrator. Utilizing the School’s research network, guidance on the direction and implementation of the research strategy was provided by a non-funded multidisciplinary steering group composed of academics and clinical academics from across the university and local trusts. The School also benefited from support from central university teams in key areas such as funding applications, marketing and ethics approvals.

Crucially, the successful NIHR NMSIDI bid provided external funding for further core research infrastructure – specifically, an IAT Project Manager and IAT Administrator. These roles provide vital operational support for the growing undergraduate and postgraduate research opportunities. Further leadership support for governance and growth of these opportunities was provided by two internally-funded leadership posts, namely an IAT Lead and an Undergraduate Research Lead. The extended research network was also used to embed voluntary Clinical Academic Training mentors, as an impartial contact point for ACFs, as well as providing careers guidance.

As more mature research groups evolve into formal centres at the School, these centres are beginning to develop their own infrastructure. For example, within the Primary Care Research Centre (PCRC), a role funded externally by the NIHR Research Capability Fund (RCF) has been established to strengthen university–primary care partnerships [33]. In addition, the university has funded a dedicated post to support the operational and collaborative mobilisation of emerging research centres.

In the early phases, the School utilized internal university funding to support essential research leadership and administration and drew on the expertise of central research support services to coordinate and manage the School’s growing portfolio of research opportunities. As these activities expanded it became clear that a more formalized and sustainable infrastructure was needed.

The inclusion of infrastructure funding as part of the NMSIDI NIHR bid acknowledged the administrative capacity required to support research engagement amongst new medical schools. The infrastructure funding provided a critical foundation to support the growing research activities. This growth has been further supported by the university through the appointment of more leadership infrastructure.

Beyond the initial NMSIDI and internal seed funding, there is a clear recognition at ARU that research activity must transition towards self-sufficiency. Future sustainability and growth of the research infrastructure will depend on securing external funding streams.

## Discussion

This case study set out to address the research question: *how can health research capacity and capability be developed in geographies historically underserved by research, in pursuit of a more equitable research ecosystem?* Our findings indicate that it is possible to establish sustainable research infrastructure in such settings through a deliberate, phased approach that combines local initiative with targeted national support. In practice, this involved designing and implementing a structured strategy for building supervisory capacity and using this as the foundation for securing external funding aimed at long-term growth and sustainability (Fig. 3).

A first key finding is the importance of developing supervisory capacity. As seen in other under-resourced contexts [34], ARU’s approach relied not on a large existing pool of senior researchers but on networked and collaborative arrangements that gradually evolved into structured groups and centres. This model enabled the School to create supervisory pathways for both undergraduate and postgraduate projects, laying the groundwork for future research leaders. Supervisory growth was not only a precondition for research expansion but also an outcome of it.

Our analysis also highlights the role of undergraduate students as the foundation of the clinical academic pipeline, and the future of clinical research. Embedding evidence-based medicine and research SSCs across the MBChB programme ensured that every student engaged meaningfully with research. This is consistent with wider literature from established medical schools showing that early exposure fosters research literacy, shapes professional identity and increases the likelihood of pursuing clinical academic careers [35]. Importantly, ARU draws much of its intake from the local area, with many graduates remaining in the region post-qualification. This reinforces the strategic value of local research opportunities in settings such as new medical schools, as the skills and confidence developed are more likely to be reinvested in underserved local health systems.

Equity was a guiding principle throughout the development of undergraduate research opportunities. By embedding research within the curriculum, opportunities were made universally accessible, reducing reliance on extracurricular participation that often favours more privileged students. This is especially relevant for students from widening participation (WP) backgrounds, many of whom juggle part-time work or financial responsibilities alongside their studies. Nationally, it is recognized that extracurricular research can be financially prohibitive, which creates inequities in who can access the benefits of early academic exposure [36]. At ARU, the availability of NIHR-funded research stipends through the New Medical Schools Intercalated Degree and Internship (NMSIDI) programme has been pivotal in addressing these barriers, enabling a diverse cohort of students to participate without financial penalty. Supporting diversity in research opportunities is not only an equity imperative but also a strategic one: graduates who reflect their local communities are better positioned to research and address local health disparities [37].

A critical enabler has been the role of targeted national funding schemes focussed on research infrastructure. Bespoke funding for new medical schools in the NIHR NMSIDI bid, and inclusion of new medical schools in the NIHR Integrated Academic Training (IAT) scheme was transformative for ARU, both practically and symbolically. These schemes reflect the NIHR’s “levelling up” agenda [38], which recognizes the need to rebalance research funding geographically to improve equity in health outcomes. Without success in these funding schemes, ARU would not have been able to develop inclusive research opportunities for medical students, nor create posts with funded protected time for postgraduate local clinical trainees, both of which are essential for reducing barriers to research engagement during clinical training, and the development of the future clinical academic workforce along with the growth in local clinical research this brings. Crucially, the team would also have lacked the administrative capacity needed to support trainees and ensuring quality of their research experience. Success in these bids and the resulting growth in research capacity and capability has further enabled ARU to secure additional internal investment and a further external funding.

This case also illustrates how a collaborative approach and foundational institutional investment, however modest, can position newer medical schools to compete effectively for external support.

Nevertheless, challenges remain. Infrastructure development is resource-intensive, and newer medical schools lack the economies of scale of established research-intensive institutions. Initial progress at ARU relied heavily on internal seed funding and voluntary expertise. While NIHR infrastructure funding has provided a critical baseline, long-term sustainability depends on embedding research support into institutional strategies and securing ongoing external investment, including exploring industry and the commercial sector, as well as expanding on established networks for continued collaborative research growth.

Taken together, these findings point towards a positive feedback loop (Fig. 4). Equitable access to research opportunities builds a diverse cohort of future clinical academics in areas underserved by research and with health inequalities. As well as increasing local research capacity this helps to improve clinical workforce attraction and retainment. Over time as supervisory expertise and research outputs grow, institutions become more competitive for external funding, which in turn strengthens infrastructure and further broadens access. Over time, through research focussed on healthcare needs of the local population, and the benefits to the local workforce, research equity can lead to a reduction in health inequalities.

This single centre case study contributes to the wider literature by showing that new medical schools can act as engines for research equity when strategies are intentionally designed around clinical academic training, collaboration, inclusivity and local relevance. Whilst centred around doctors, the principles illustrated of phased supervisory development, curriculum integration, targeted use of levelling-up funding and prioritization of widening participation, are transferable to other underserved geographies and healthcare professions. By adopting such approaches, medical schools, universities, funding bodies and health systems can work together in taking concrete steps towards redressing inequities in research capacity and capability, ultimately supporting improvements in population health outcomes where they are most urgently needed.

## Limitations

This single-institution case study is context-specific and transferable rather than generalizable. Use of institutional documentary data limits insight into individual experiences and causal inference, and the focus on medical education may reduce applicability to other professions. Early progress relied on seed funding and time-limited schemes, with sustainability yet to be established. Downstream impact was not evaluated; future work could quantitatively and quantitatively assess effects on research participation, workforce retention, clinical academic progression and local health inequalities.

## Conclusions

Levelling the research funding landscape is essential if we are to make meaningful progress in reducing health inequalities [38]. Smaller, emerging clinical research centres, particularly those based in historically underfunded regions, often lack the infrastructure and resources needed to initiate and sustain research activity. This is largely due to a funding system that rewards established track records, creating a cycle where those with the least capacity are least able to compete [39].

This case demonstrates that as well as a collaborative approach, targeted investment is needed to provide these centres with an initial foothold, especially in the form of infrastructure funding. This foundational support enables them to harness and coordinate existing expertise, build supervisory capacity and begin generating the outputs necessary to compete for larger grants. Such an approach is critical not only for achieving equity in research funding, but also for creating a sustainable and locally grounded model of research that can meaningfully address local population health needs and address health inequalities.

## Supplementary Information

Supplementary Material 1.

Supplementary Material 2.

Supplementary Material 3.

## Data Availability

No datasets were generated or analysed during the current study.

## Abbreviations

ACF: Academic Clinical Fellowship
DHSC: Department of Health and Social Care
EBM: Evidence-Based Medicine
IAT: Integrated Academic Training
MBChB: Bachelor of Medicine, Bachelor of Surgery
NHS: National Health Service
NIHR: National Institute for Health and Care Research
NMSIDI: New Medical Schools Intercalated Degree and Internship
PCRC: Primary Care Research Centre
PI: Principal Investigator
RCF: Research Capability Fund
RCP: Royal College of Physicians
SFP: Specialist Foundation Programme
SSC: Special Study Component
UKRI: UK Research and Innovation
WP: Widening Participation
