# Supplementary material for: Developing health research capacity and capability in underserved geographies: a case study from a new medical school
Source: Health Res Policy Syst. 2026 Mar 9;24:36. doi: 10.1186/s12961-026-01452-x (PMC13085571; doi:10.1186/s12961-026-01452-x)
Supplement: Supplementary file 1 — Supplementary Material 1. [file 12961_2026_1452_MOESM1_ESM.pdf]

Broad research theme	Narrow research theme
Medical education	Transition
	Medical curricula
	GP training
	Peer-peer/near-peer learning
	Inequalities in education
	Simulation
	Assessment for learning
Anatomy	Neuroanatomy
	Embalming
	Ultrasound in anatomy
	Functional anatomy
	Ethics in anatomy
Machine learning / artificial intelligience	Machine learning / AI
	Robotic surgery
Mental health / behavioural science	Staff resilience
	Risk-taking behaviour
	PTSD
	Mental health impact of conflict
Population health	Disaster mitigation
	Obesity in CVS disease
Infection	HIV transmission
	Early detection of infection
	Antimicrobial stewardship - prescrib
	Bone infections
Cancer	Cancer service
	Cancer biomarkers
	Skin cancer treatment delays
	Breast reconstruction
	Biomarkers
	Drug targets
	Gene regulation
Primary care	GP training
	Health economics
	Improvement science technologies
	Primary care support models
	Health inequalities in primary care
	Inclusion health
	Primary care workforce
Health services research	Impact of online / hybrid consultati
	Health economics
	Improvement science technologies
Impact on patients / families	Traumatic injury
	Living with disability
	Chronic pain
	Supporting carers

	Narrative practice
**Development / paediatrics**	Sleep in children
	Neurodevelopment
	Lung development and disorders
	Genetics in cerebral palsy
	Paediatric surgery
