# Supplementary material for: Developing health research capacity and capability in underserved geographies: a case study from a new medical school
Source: Health Res Policy Syst. 2026 Mar 9;24:36. doi: 10.1186/s12961-026-01452-x (PMC13085571; doi:10.1186/s12961-026-01452-x)
Supplement: Supplementary file 2 — Supplementary Material 2. [file 12961_2026_1452_MOESM2_ESM.pdf]

October 2022

Developing research capacity and capability across the School of Medicine

Authors: Jo-Anne Johnson, Sanjiv Ahluwalia

Introduction

This paper sets out an emergent approach to the development of research capacity, capability and a diversity of skill base across the School of Medicine (SoM). In doing so there are several principles that are assumed/articulated:

- Collaboration in high quality research is at the heart of achieving aspirations for developing a sustainable research and innovation ecosystem
- Research themes and programmes are based on the needs of local populations/services, national and international challenges mapped onto the interests and skills of (groups of) researchers
- Clarity on roles and responsibilities in relation to developing a research infrastructure is essential
- Research is finance-intensive, and sustainability needs to be considered
- Developing a pipeline of research talent is key to success, from undergraduate through to post-doctoral journeys and beyond
- Distinctions between research, evaluation, and implementation are best recognised but should not become a barrier to support infrastructure and talent development
- Successful research infrastructure development requires multi-professional and expert (in research development) input with requisite resources

This paper charts current activity underway in support, emerging/evolving relationships, areas where further focus is required, and proposed implementation steps.

Background

The SoM was established in 2018 to deliver undergraduate and postgraduate teaching programmes. The focus of activity has been on meeting the expectations of regulators and stakeholders in delivery of these educational programmes. As we enter the fifth year of existence, our attention is now turning to developing an active research programme that enriches and supplements the educational activity of the SoM. This ambition is in keeping with the stage of development associated with Medical Schools of such short duration. It is also in line with the national drive (MSC, NIHR) to re-distribute clinical research funding to areas of the population with greatest healthcare needs. These areas are typically where the new medical schools across the UK are situated and ARU, as one of the leading medical schools in the 2018 medical schools group, is at the heart of this strategy.

Aims

- Establish, promote, recruit to and support a clinical academic training pathway
- Attract and retain medical students / trainees in Essex – serve local population need
- Create a research positive environment for staff, students and trainees
- Build students and trainees as clinical researchers and staff in SoM as supervisors
- Use collaborations with research active units across HEMS, the broader University and externally to form supervisory capacity/capability

- Use strengths and expertise within the SoM to develop research themes within the SoM which compliments existing research in the wider faculty, aligns with the university research strategy and focusses on local population needs
- Ensure sustainability of the SoM research activity longer-term
- Step towards multiprofessional and interdisciplinary research (longer term focus on integrated training for non-clinicians)

Current activity

The SoM, following COVID, has initiated the process of developing research capability and capacity through a range of activities as articulated below.

- Developed a research strategy steering group to inform direction and implementation of the researchstartegy
- Identified research groups that relate to existing research strengths, each with a research group lead, in varying stages of development
- Developed relationships with research units outside the medical school, and other medical schools (ongoing)
- Developed incentives for encouraging research participation amongst medical students

Future activity

The National Institute for Health Research (NIHR) and Health Education England (HEE) have developed a formal programme of developing clinical academics through provision of funded placements (within universities) at all stages from foundation, through higher training, into early post-doctoral programmes. In line with the national incentive to push research funding into deprived areas, is the drive to provide clinical academic training posts to new medical schools.

The main focal point of the SoM research strategy is the development of a clinical academic training pathway. As well as attracting high-calibre medical students / trainee doctors to Essex, this pathway will generate a pipeline of local clinical academics. Many of these clinical academics will remain in the Essex region, conducting clinical research targeting local population need, including deprived areas (e.g. costal towns) which have historically been neglected. They will also feed back into ARU SoM from an education perspective.

The focus of our research strategy shorter-term is on the establishment of Academic Foundation Posts (AFPs) and Academic Clinical Fellowship (ACF) posts at ARU SoM.

Background to AFPs and ACFs

AFPs (research):

AFPs (soon to be called Specialist Foundation Posts) are fully-funded 2-year foundation trainee posts with 2 x 4 month blocks in research (2 x 8 months clinical). These posts are funded and allocated annually by the Deanery who manage the recruitment and liaison with local trusts regarding the clinical placements associated with these posts.

October 2022

ARU SoM already have a number of allocated education-focussed AFPs. The SoM have been successful in bidding for 12 research-focussed AFPs to start August 2024, and from a research placement perspective, will be placed in VERI, VFI, the circulatory health research group and St Andrew's burns and plastics/ARU research group (StAAR). The SoM Research Lead will coordinate the program alongside HEE.

ACFs

ACFs are allocated annually to integrated academic training partnerships – between the HEE local office or NIHR, the HEI and an NHS organisation.

Posts are 3 years in duration (4 years GP), with 25% of this time in academic training, and 75% in clinical training. The academic training does not need to be associated with a Master's degree, but the offering of some Master's modules is expected. Recruitment of candidates to ACF posts is managed by HEE local office/NIHR.

We are bidding for 2 NIHR ACFs in January 2023 (for September 2024 start)

Establishing a Clinical Academic Training Pathway

At the heart of our bids for clinical academic training posts is the establishment of supervisory capacity and research capability at the SoM. In order to achieve this, a range of actions developed in parallel are required. Their cumulative effect is likely to be complementary.

- Research development opportunities for students

The clinical academic training pathway starts at undergraduate level. Inspiring the next generation of clinical academics starts at medical school, as does promoting evidence based practice. An early activity is to formalise opportunities for capability attainment in clinical research amongst SoM learners. We have already made substantial advancements here, with the percentage of students taking an intercalated degree sitting at 10-15%, which compares favourably with other new medical schools.

We have also introduced 'evidence-based medicine' as a core component of the undergraduate curriculum, journal clubs and research seminars with opportunities for students to showcase their work. We have also introduced a £20,000 annual bursary which medical students can apply to in order to present their work at conferences. There is a joint bid underway with the University of Cambridge University for a Wellcome Trust INSPIRE grant intended to fund additional activities to encourage research participation amongst medical students. Finally, our supervisor – student matching scheme has seen over 60 medical students actively involved in research in their own time across the university.

- Developing research groups

The established research groups continue their journey to maturity. The aim is to encourage a focus for the research groups which aligns with expertise and unique selling points of the SoM, with the ARU Research and Innovation Strategy, with the RII research themes and the NIH-Wide Strategic Plan, and with a focus on local population need. Working with research support units within ARU (e.g. Doctoral School, RIDO), the groups will be encouraged to applying for grants, and be supported in higher degree attainment, as well as contributing to knowledge exchange. The SoM research groups will me mentored by established researchers within ARU (e.g. Allied Health and VERI).

- Expand collaborative networks

The research groups have an open approach to membership based on shared interests and complementary skills base. This has generated relationships with research active units across ARU with NHS Trusts and other Universities (e.g. University of Cambridge). These links are vital in demonstrating supervisory capacity and through joint supervision will develop supervisory experience at the SoM. This will be key to our success in bidding for clinical academic training posts now and in the future. Further expansion of these groups through engagement with external stakeholders (beyond HEMS and ARU) is likely to increase the opportunities for research funding applications.

- Innovation

ARU has developed a research and innovation strategy (2022) that supports a strong link between research and translation into practice. The SoM has a strong link with the ARU innovation team through Professor Tony Young. It will be our intention to use emergent technologies research (e.g., TIERS (The Institute of Excellence in Robotic Surgery)) to explore opportunities for building research translation capabilities in the medical arena. This in turn would provide further research opportunities for medical students / clinical trainees.

<u>Governance and infrastructure</u>

This work is governed through a steering group consisting of the following team members, who meet on a 6-monthly basis:

- Research Lead, SoM (Chair)
- Head of SoM
- DDRI, HEMS
- Deputy Head of School PG, SoM
- Representation from MTRC
- Representation from VFI
- Representation from VERI
- Head of School Allied Health
- Director of Synapse Centre for Neurodisability, ESNEFT

October 2022

- St Andrews & ARU research group lead
- Representation from MSE FT

Below is a diagram outlining the proposed SoM research infrastructure.

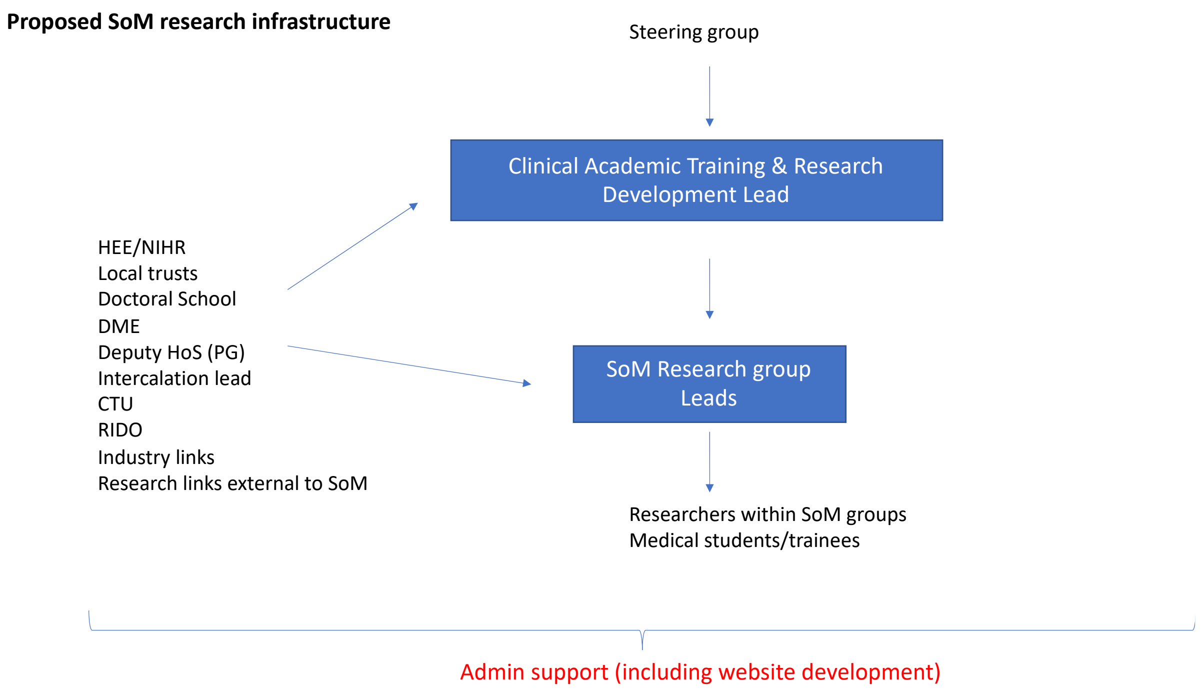

<u>Outcomes</u>

<u>Short-term (0-2 years)</u>

- Recruit to the role of research lead to develop the SoM research infrastructure, including the appointment of new / inclusion of existing clinical researchers affiliated to the SoM
- Establish the SoM research infrastructure including links to the wider faculty
- Establish evidence-based medicine theme in the medical undergraduate curriculum
- Apply for INSPIRE grant (joing bid with Cambridge University) to support ideas for engaging medical students in research
- Using existing collaborative network, put joint supervisory bids in for 12 x AFPs and 2 x ACFs in the first year
- Utilise mentorship / expertise outside the SoM to expand research groups and develop a focus for each research group
- Expansion of the SoM research & innovation collaborative network (internally and externally)
- Development of a School of Medicine research group website
- Begin development of SoM Master's courses (e.g., MRes, Masters in Surgical Robotics)
- Reach out to wider faculty/faculties outside HEMS for inclusion of existing BSc/MSc courses as intercalated degrees for internal and external medical students

- Researcher development training for grants and publications

Intermediate term (3-5 years)

- Success in the bidding for PhD/ProfDoc/MD Studentships
- Success in attracting self-funded PhD/ProfDoc/MD Studentships
- Define contribution to research, impact and knowledge exchange (REF/KEF)
- Complete development of and introduce Masters' degrees
- Bids for Academic Clinical Lectureships
- Diversification of external grant applications and success in funding awarded
- Strengthen regional research collaborations
- Establish research links with national and international medical schools
- Planning for introduction of MBPhD

Longer term (>5years)

- Form further industry links for innovative projects
- Strengthen national / international research collaborations
- Demonstrate exemplary retention and completion rates for PGR students
- Re-investment to expand research infrastructure
- Introduce MBPhD into undergraduate medical program

October 2022

Appendix 1 Proposed roles and responsibilities

Clinical Academic Training & Research Development Lead (SoM Research Lead)

Supported by Steering Group

- Form a SoM infrastructure which links in with the wider faculty (see diagram above)
- Regularly review outcomes ensuring timepoints are met
- Lead on bids for and implementation of clinical academic training posts
- Support research groups and their leads in their remits as described below
- Provide strategic direction for establishment of the SoM as a research active educational unit
- Form external collaborations within and outside ARU
- Provide clinical leadership for the expansion of the CTU
- Design/implementation of taught longitudinal theme of evidence-based medicine in the undergraduate medical curriculum
- Lead on the development of Master's research degrees and the MBPhD program
- Lead on intercalation
- Operational management of the developing UG/PG clinical academic training pathway

SoM Research Group Leads

Supported by Clinical Academic Training & Research Development Lead

- Under mentorship of the Clinical Academic Training & Research Development Lead and established researchers external to the SoM, establish a direction for their research group which:
    - Fits with the ARU research and innovation strategy
    - Fits with the NIH-Wide Strategic Plan
    - Focuses on local population needs
    - Focuses on the expertise of the SoM
- Use internal and external collaborations to expand their research group membership
- Supports the development of impactful but achievable research projects within their team
- Works with RIDO in promoting and supporting grant applications and studentship applications amongst their group
- Form links with industry for any applicable projects within their group
- Generate research projects (including collaborative projects) within their team which attract funded clinical academic trainee posts and studentships
- Utilise collaborations to encourage joint supervision of research projects in order to promote supervisory capacity within their group
- Promote the career development of researchers within their group (e.g. higher degrees / leadership experience)
