# Supplementary material for: Developing health research capacity and capability in underserved geographies: a case study from a new medical school
Source: Health Res Policy Syst. 2026 Mar 9;24:36. doi: 10.1186/s12961-026-01452-x (PMC13085571; doi:10.1186/s12961-026-01452-x)
Supplement: Supplementary file 3 — Supplementary Material 3. [file 12961_2026_1452_MOESM3_ESM.pdf]

August 2025

ARU School of Medicine Research Strategy v4

Why is growing research at the School of Medicine important?

It is well established that healthcare trusts and practices engaged in research achieve better patient outcomes. Research involvement also enhances staff recruitment and retention, further strengthening local healthcare provision. Yet nationally, there has been a steady decline in the number of clinical academics. Ensuring that clinicians are both engaged in and leading clinical and translational research is critical, as this grounds research in real-world clinical need.

The School of Medicine is in a unique position to contribute to reversing this trend through access to NIHR Integrated Academic Training (IAT) posts and Specialist Foundation Programme (SFP) posts. These clinical training roles, which incorporate protected time for research, have the potential to attract clinicians to the region and are essential for building a pipeline of research-active and research-informed practitioners. Many of these individuals are likely to remain in Essex, supporting the development of a sustainable, locally embedded research ecosystem.

In contrast to other research centres within the university, our research strategy places clinical academic training—at both undergraduate and postgraduate levels—at its core. By focusing on developing and retaining a research-informed local healthcare workforce, our goal is to directly improve health outcomes for the populations we serve in Essex.

Looking back over the past year

Over the past academic year, our focus has been on recruiting and supporting the increasing number of IAT and SFP trainees, while also strengthening and embedding our research infrastructure and governance. In parallel, we have been driving forward the development of our own research within the School.

Clinical Academic Training

Over the past academic year, we have successfully recruited to our four NIHR ACF posts across the clinical specialties of primary care, psychiatry, and ophthalmology (Appendix A), with research placements hosted in university institutes of excellence including the Veterans and Families Institute (VFI, ARU) and the Vision and Eye Research Institute (VERI, ARU). In addition, we recruited to one of two locally funded ACF posts within the Virtual Imaging Academy (VIA; ARU–NHSEngland–NNUH) and are currently in the process of recruiting to the second.

We have also been successful in our most recent NIHR IAT bid, securing a further four ACF posts for 2026/2027. These posts broaden the range of opportunities available, while continuing to be centred on research excellence within the university. Research areas include the Cambridge Institute of Music Therapy Research (CIMTR); social sciences research within VFI, the International Policing and Public Protection Research Institute (IPPPRI), and the Centre of Excellence for Equity in Uniformed Public Services (CEEUPS); the Primary Care Research Centre (PCRC) at the School of Medicine; and the ARU Biomedical Research Group (FSE).

Alongside this expansion, we have embedded a comprehensive IAT training programme, developed supportive infrastructure for both supervisors and trainees, and introduced a combined staff/trainee ACF handbook.

In addition we recruited a further12 SFPs (Appendix A) who are embedded within VERI, the VFI, the cardiothoracic Centre (CTC) Basildon, inclusion health research group, ARU and burns&plastics (Broomfield). We will be introducing an SFP to the PCRC from August 2026.

Research at the School

Our research activity at the School has expanded in parallel with the growth of IAT training, most notably through the approval of the Primary Care Research Centre (PCRC). The PCRC now has dedicated infrastructure, including administrative support and a jointly funded partnership role between ARU and MSEICB.

The Centre of Excellence in Robotic Surgery (TIERS) has continued to deliver research outputs and has welcomed its first MSc students. TIERS has also broadened its scope by forming a new collaborative research group with partners across the East of England, with the strategic aim of developing funding bids and securing future IAT trainees.

The Medical Education Research Group has expanded its network of internal and external collaborators and has been incorporated under the PCRC governance structure to ensure stability and support as it develops. Similarly, the Anatomy Research Group continues to build momentum and has been integrated into the TIERS collaborative research group, where synergies and shared infrastructure provide a strong foundation for growth.

Our collaboration with the Cardiothoracic Centre (CTC), Basildon, also continues to strengthen. The School of Medicine funds research time for and line manages four clinicians within the CTC. The CTC in return is increasingly involving our students and trainees in its research activity, contributing to both their development and the centre's outputs. In recognition of our support, the CTC acknowledges the School in its research outputs. The CTC consistently produces high-quality research and has now started to bring in grant income.

The Vision and Eye Research Institute (VERI), one of the university's designated institutes of excellence, remains housed within the School of Medicine. VERI operates with its own infrastructure, governance, and reporting processes, which sit outside the scope of this strategy. Nonetheless, we continue to collaborate closely with VERI, which provides research placements for our students and trainees and plays an important advisory role on our research steering group.

As we build our research activity, we have adopted a strongly collaborative approach, for example academics from multiple faculties are members of the PCRC, reflecting the strong synergies between their work and the priorities of primary and community care. We have achieved this by drawing on the networks established through our spread of IAT and undergraduate trainee placement opportunities. This approach to IAT and undergraduate training not only enhances the School's reputation in the IAT and research space, but also provides a valuable resource for research groups across ARU.

Research infrastructure

In order to strengthen our research infrastructure (Appendix B), the School has appointed an IAT Lead alongside NIHR-funded Project Manager and Administrator posts. Together, this team has established a robust governance framework with defined reporting lines and responsibilities. At the operational level, this includes the Essex Clinical Academic Training office (ECAT) as well as support from the university's research services. In line with our growth and evolving strategy, our meeting structures have been re-purposed, each now operating with clear membership, and Terms of Reference.

We have continued to grow our research network, both within the wider university, as well as within the clinical community, within research networks such as the RDN, and with other universities. These collaborations are important in terms of growing our own research, but also providing opportunities in research development for our trainees and students.

Undergraduate research

Our numerous formal and informal undergraduate research opportunities have been consolidated in the first iteration of the Undergraduate Research Guide, which maps a five-year journey of available research experiences which include as well as run alongside the core curriculum. As we approach the final year of NIHR-funded undergraduate opportunities (intercalations and internships – Appendix C), we have experienced challenges in filling the locally funded intercalation places. This possibly reflects a national trend of declining numbers of intercalating students, driven by factors such as financial pressures and the removal of intercalation from foundation programme application requirements.

Looking ahead, and in line with other medical schools, we are reviewing our entry criteria for intercalation with the intention of making them more flexible, while retaining the requirement for students to have passed all summative assessments up to the point of entry. We are also extending eligibility to include entry from Year 4.

Although intercalating locally offers financial advantages, many of our students continue to intercalate externally. At present, 11 ARU courses are accredited for intercalation, though the majority sit outside the School of Medicine. Within the School, we now have one master's course accredited, and our ambition is to expand this offer by developing and accrediting further SoM courses for intercalation in the coming years.

Looking ahead to the next academic year

Our priority for the coming year is sustainability. To maintain and support our expanded infrastructure, we now need to focus on generating research income. This will be achieved by strengthening and growing research within the School, with particular emphasis on our centres and institutes of excellence — namely the PCRC and TIERS — as well as key affiliated partners such as the CTC.

In house clinical academic training opportunities
As highlighted, we remain reliant on expertise outside the School to drive much of our research activity, but we also have the opportunity to harness the potential of SFP/IAT trainees and medical students to strengthen and grow our research base, with priorities aligned with the School's overarching research aim – to improve the healthcare outcomes of our local population. To date, our IAT trainees have been placed exclusively in external groups. This has been highly beneficial in terms of securing our first allocations, building our reputation, and establishing a supportive research network.

Our focus for the next year is to bring our in-house research to a stage where we can successfully embed postgraduate research trainees and medical students, ensuring they receive a high-quality research experience. The foundations for this are already in place — for example, one of the ACFs from the 2025 NIHR is bid due to start in the PCRC in August 2027. It is vital that strong research activity is available for this trainee to engage with.

The prospect of incoming trainees provides a powerful incentive to advance our research to the next stage — including readiness to apply for external funding, with provision for research infrastructure built into these bids. Once embedded, trainees and students will in turn accelerate the growth of research outputs, strengthen future funding applications, and expand our capacity to support further cohorts.

Intercalation
We will review both the eligibility criteria and the local intercalation opportunities available, with the aim of encouraging more students to take up this invaluable pathway. Intercalation is designed to

inspire students to pursue a research-active career within the local area, and this review will ensure we make best use of the final year of NIHR funding in this space.

Innovation and entrepreneurship
Over the past two years, we have introduced a nine-month, year four longitudinal Special Study Component (SSC) with pathways in research, quality improvement (in collaboration with the MSE Quality Improvement Office), and entrepreneurship & innovation/digital transformation (in collaboration with the CEP, ARU, and MSEFT).

Uptake of the entrepreneurship & innovation/digital transformation pathway has been limited, despite the introduction of a dedicated Innovation Day in year 3. To address this, we are working with the University's Student Enterprise and Entrepreneurship team to encourage greater student engagement. This includes piloting an *Entrepreneurship Champion* role, who will collaborate with the School Research Lead and the Year 3 Innovation Day Lead to review and strengthen our offerings in this space, as well as serve as a direct point of contact for students.

Looking forward, we are also exploring ways to extend and embed our innovation strategy more widely across the Faculty.

Marketing
We have successfully secured dedicated websites for both the PCRC and TIERS. Over the coming year, our priority will be to develop broader webpages to showcase research across the School of Medicine as a whole.

Governance
As our research activity and student/trainee opportunities have grown, the number of meetings has also increased. With our expanded team, we will now review the membership and structure of research meetings, with the aim of streamlining and improving efficiency.

REF29
As noted in the previous strategy, challenges remain around reporting funding streams and attributing academic outputs within collaborative groups such as ours. This issue has been flagged to the university and is currently under review. The income from our NIHR bids is unique to the university in that most of the monetary income does not flow through the university but directly to the salary costs of the individual concerned. We are actively looking at ways that this income can be recognised by the university as benefit in kind.

In the meantime, we will continue to monitor and record our outputs, funding streams, and impact at the level of centres, staff, students, and trainees, using this data to inform reporting into faculty committees such as FRIC (Appendix D).

In addition, the PCRC will contribute to the REF29 convener group for HEMS, ensuring that our work is represented in university-wide research assessment processes.

Longer Term Objectives

Building on our existing partnerships with local trusts, we aim to establish further joint SoM–trust research centres, modelled on the successful collaboration with the CTC. These centres would provide trusts with access to the School's supportive research infrastructure, the university's research support services, and academic expertise, as well as opportunities for student and trainee involvement in clinical research. For the School, this approach will both strengthen our research

portfolio and reputation, and expand the range of clinical research opportunities available to our students and trainees.

As our collaborative network grows, several research themes continue to emerge organically, reflecting both local and academic interest. These could provide the basis for new research groups, and centres, initially developed through existing infrastructure — for example, paediatrics and child health within the PCRC.

A longer-term goal is also to support our existing smaller in-house groups, such as anatomy and medical education research, to develop into centres in their own right. Achieving this will require sustained internal and external expertise, as well as successful external grant bids and the creation of dedicated infrastructures. Embedding these groups within more established centres at this stage provides a vital foundation for their future growth.

Appendices

Appendix A – Summary of Clinical Academic Trainees

ACFs	Specialty	Research Team	Start date	Funding
1	General Practice	Veterans and Families Institute	Aug-24	NIHR
1	Ophthalmology	Vision and Eye Research Institute	Mar-25	NIHR
1	Ophthalmology	Vision and Eye Research Institute	Aug-25	NIHR
1	Psychiatry	Veterans and Families Institute	Aug-25	NIHR
1	Clinical Radiology	Virtual Imaging Academy	Aug-25	Locally Funded

**Specialised Foundation Programme Trainees**	
**Research SFPs**	
FY2 (August 2024 start)	11
FY1 (August 2025 start)	12
**Medical Education SFPs**	
FY2 PG Cert	12

**Total Clinical Academic Trainees currently in post**	
ACFs	5
Research SFPs	23
Medical Education SFPs	12
Total	40

Appendix B – School of Medicine Research Infrastructure

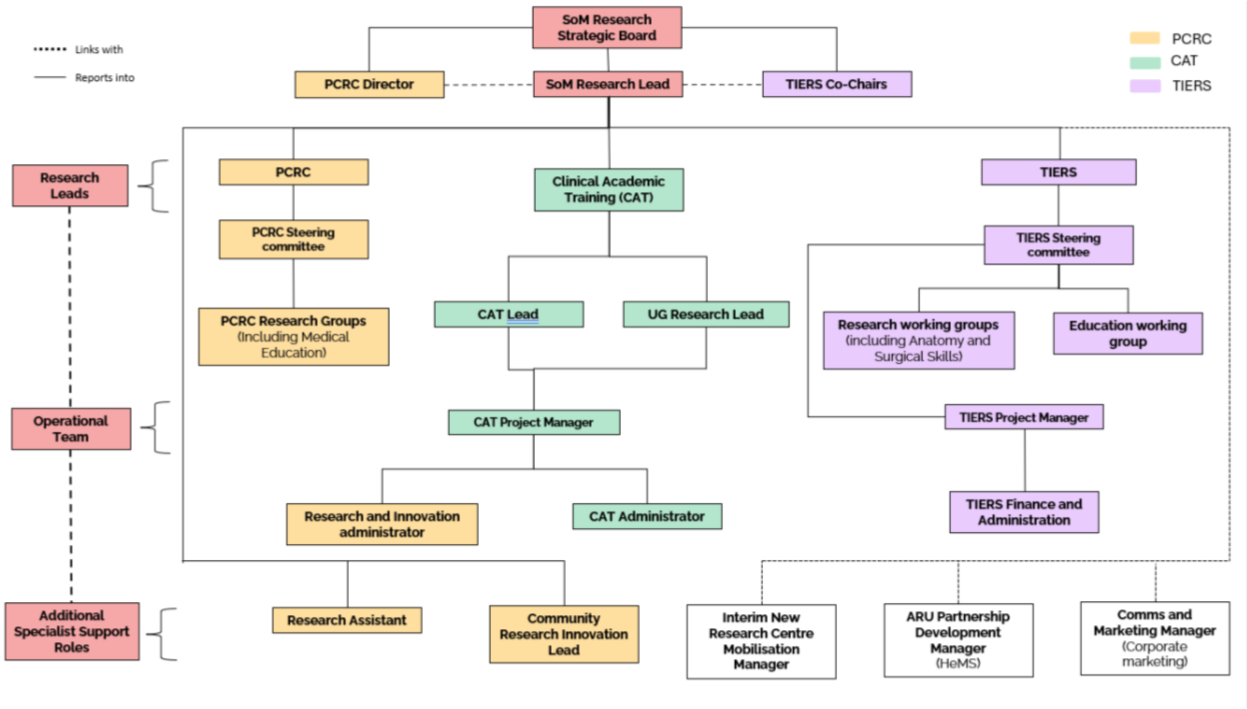

Appendix C – Undergraduate medical students

Undergraduate research summary:

UG	AY 23/24	AY 24/25	AY 25/26
Internships	11	15	
Intercalation		5	10
Year 4 SSCs		113	113
UG Conference Funding	20	57	

Intercalating medical students overview:

**Intercalation Year**	**Total**	Internal - NIHR Funded	External
**24/25 AY**	5	4	1
**25/26 AY**	10	4	6

Intercalating medical students allocated AY 2024/25, intercalating 2025/26

Year Group	University	Course
Year 3	ARU	Robotic Surgery
Year 4	ARU	MSc Robotic surgery (First Choice)
Year 4	ARU	MSc Mental Health - ARU
year 3	ARU	MSc Child and adolescent mental wellbeing
Year 3	University of Liverpool	Acute, Critical and Emergency Care BSc Hons - Liverpool University
Year 3	King's College London	Primary Care BSc - King's College London
Year 3	Imperial College London	Medical sciences with management iBsc- Imperial College London
Year 3	Queen Mary University of London	FT Laparoscopic Surgery and Surgical Skills MSc - Queen Mary University of London
Year 3	University of Edinburgh	Clinical Anatomy MSc - THE UNIVERSITY of EDINBURGH
Year 3	University of Cambridge	MPhil in Obesity, Endocrinology and Metabolism

NIHR funded undergraduate research internships overview

SoM Internships		*Notes*
**23/24 AY**	11	*(June - August 2024. Note, 10 NIHR Funded, 1 SoM Funded)*
**24/25 AY**	15	*(June - August 2025)*
**25/26 AY**	20	*Upcoming 2026*

NIHR funded undergraduate research internships allocated summer 2025

Internship Title	Primary supervisor	Second supervisor	Internship Host	No of internships	Appointed Student Year Group	No Of Weeks
Clinical Data Project Essex Cardiothoracic Centre	Thomas Keeble		CTC	1	Year 2	10 Weeks
Service Evaluation of Long-Term Outcomes of Through-Knee Amputation	Ankur Thapar		CTC	1	Year 2	10 Weeks
Healthcare providers perspectives on the impact of assistance dog retirement on user wellbeing	Nieky van Vegel	Hilary Engward	Veterans and Families Institute for Military Social Research	1	Year 2	10 Weeks
Management of Anaemia in cancer patients	Rafiqul Islam	Nicol George	ARU-Chelmsford	1	Year 3	6 Weeks
Scoping Study investigating Annual Health Checks for	Catherine Kennelly	Margaret Greenfields	HEMS	1	Year 4	6 Weeks

People with Learning Disabilities						
A wearable muscle myography-based armband for upper limb gesture recognition	Shabnam Sadeghi Esfahlani	Dan Robbins	FSE, EBE	2	Year 4 + Year 3	6 Weeks
Assay development to detect Alzheimer's disease biomarker	Ling Hwang	Dan Robbins	School of Medicine/ MTRC	2	Both year 3	6 Weeks
Creation and documentation of human prosections of use in teaching and research	Adam Raven	Vicky Wilson	School of Medicine - Anatomy Centre	2	Both Year 1	10 Weeks
Understanding the unmet nutritional needs of stroke survivors	Sanjoy Deb	Alex Lang	School of psychology, sport and sensory science	2	Both year 3	6 Weeks
The impact of being a member of a specialist community for those affected by brain injury.	Sarah McLachlan	Nora Brennan	CEEUPS	2	Year 1 + Year 2	10 Weeks

Year 4 SSC overview

Year 4 SSC	23/24	24/25	25/26
Research Projects	74	80	88
Quality Improvement Project	27	22	10
Entrepreneurship and Innovation Project	8	9	14
Digital Transformation Project		2	1

**Total Year 4 SSC students**	109	113	113

Undergraduate research SSC project areas 2025/26

25/26 Research Project Breakdown (by Themes)

**Theme**	**Number of Research SSC students**
Anatomy	13
Basic Science & Disease Mechanisms	12
Clinical Research	10
Inclusion & Diversity	3
Medical Education	23
Mental Health	1
Public Health & Policy	6
Medical Technology & Innovation	15
Women's Health	5

Undergraduate Student Outputs:

	**23/24**	**24/25**
UG Students Allocated Conference Funding	20	57
UG Student Publications	23	21
UG Student Oral and Poster Conference Presentations*	39	72

**This includes student participation in internal ARU conferences, such as the School of Medicine Research Conference, to explain why there is a difference between the number of students allocated conference funding and the number of students actually giving conference presentations.*

Undergraduate research – additional information

Upcoming 25/26 Academic Year / 26/27 Financial Year	
NIHR Funded Internships	20
NIHR Funded Intercalation places	15
Student Research Forum	
Student Phase Research Lead	3

Appendix D – School of Medicine Research Funding Streams

Funding Stream	Amount (if applicable)	Description
NIHR NMS IDI	• Total 2024 – 2027: £1,429,810<br>• 25/26 Financial Year: £476,620<br>• 26/27 Financial Year: £646,080	The New Medical Schools NIHR IDI bid brings in funding for Intercalation, SoM Internships, Infrastructure staffing costs and a fund for building an Academic Society / Community
NIHR IAT Academic Clinical Fellow (ACF) funding	• £4,500 per ACF Research training costs across the 3 year programme<br>• £1,112 administration funding per ACF<br>• £1000 Conference bursary per ACF per year	25/26 Academic Year (AY) we have 2 NIHR funded ACFs currently in post, noting an additional 2 ACFs started in 24/25 AY and we have 1 locally funded ACF.
QR Funding	• £10,000 (2025-26)	Support for medical students in research, and bench fees for clinical academic trainees in research
SoM Conference Funding - Undergraduate	• £20,000 (2025-26)	Funding set aside within the SoM Budget to support UG Student attendance to National and International Research Conferences
SoM Conference Funding – SFP Trainees	• £6,000 (£250 per trainee)	Funding set aside within the SoM Budget to support SFP trainee attendance to National and International Research Conferences - £250 per Research SFP
Primary Care Research Centre		Research funding bought in from PCRC Research Groups / Staff funding bids
